# Hospital Expenditure and Efficiency

**Published:** 1905-04-15

**Authors:** 


### HOSPITAL EXPENDITURE AND EFFICIENCY.

Mr. Sydney Holland writes under date March 5th (*sic*) as follows :—

As you say that the advantage of putting your reply in juxtaposition to my letter is "thus finally to dispose of the discussion in one article," it is useless for me to send the very obvious answers to many of your remarks.

. . . . . . . . . .

You will allow me, I am sure, to add that the insulting remarks about the London Hospital management, of which I complained, appeared in the preface to the "Burdett's Annual," which came out in 1904, but which referred to the figures of 1903. Thus I called it the 1903 edition.

It is astonishing, I confess, knowing the writer of the preface and the writer of this article in The Hospital to be one and the same person, to learn, that though I quoted the exact words used, the answer should be made, "We can find no reference, insulting or otherwise, in the preface to the 1903 edition.

Yours faithfully,

Sydney Holland.

Once again Mr. Holland has fallen into error, for there is no reference to the London Hospital in the preface to "Burdett's Annual" for 1904 any more than there was in that for 1903. On page 82 of the 1904 edition, Chapter III., the following appears :—

"The more thoroughly our hospitals are inspected the more it becomes essential to the maintenance of efficiency, possibly to the enforcement of strict economy, that the administration over each hospital should be placed under the direct control of a Resident Medical Superintendent. It is very remarkable that the London Hospital, at the present time, should have no such officer in responsible charge of its management, and that the work is being carried on without any such official, who is constantly on the spot and capable of supervising every department of its work. It is tempting Providence to endeavour to carry on the affairs of the largest hospital in London with the maximum of efficiency in the absence of a resident superintendent, by whom adequate control can be exercised continuously over the whole establishment. We know full well that the present unfilled office is regarded with grave anxiety by many of the best friends of the London Hospital, and we can only hope that wiser counsels will prevail without further delay. No hospital of importance, which does not possess a system whereby continuous and adequate control can be exercised from top to bottom, can expect to be carried on without extravagance, waste, and faults of discipline, which must have a tendency to increase year by year."

If this be the reference of which Mr. Sydney Holland complains, it merely asserts a sound principle in hospital administration which is demonstrably provable to be true. There is, in fact, nothing in the statement which can be properly described as "insulting," as our readers can see for themselves.—Ed. The Hospital.

